# DDX11 interacts with PARP1 to facilitate PARylation, thereby promoting gallbladder cancer progression and conferring gemcitabine resistance: DDX11 interacts with PARP1 to facilitate PARylation

**DOI:** 10.3724/abbs.2025155

**Published:** 2025-08-26

**Authors:** Yuan Gao, Junchi Liu, Xiang Yao, Letian Gong, Shubin Luo, Chaoxian Zhao, Shaofeng Pu, Ganglong Gao

**Affiliations:** 1 Department of Biliary-Pancreatic Surgery Renji Hospital Shanghai Jiao Tong University School of Medicine Shanghai 200127 China; 2 Shanghai Key Laboratory of Systems Regulation and Clinical Translation for Cancer Shanghai 200127 China; 3 Department of Pain Management Shanghai Jiao Tong University Affiliated Sixth People’s Hospital Shanghai 200233 China; 4 Department of General Surgery the First People’s Hospital of Jinghong Jinghong 666100 China

**Keywords:** gallbladder cancer, chemotherapy resistance, DDX11, PARP1, PARylation

## Abstract

Gemcitabine resistance poses a significant challenge in gallbladder cancer (GBC) treatment, necessitating exploration of its molecular mechanisms. This study focuses on DDX11, which is highly expressed in gemcitabine-resistant GBC cells, suggesting a potential role in DNA damage repair. We establish gemcitabine-resistant GBC cell lines and observe significantly higher DDX11 expression in these cells than in parental cells. Clinical tissue analysis through qRT-PCR, western blot analysis, and immunohistochemistry confirms elevated DDX11 levels in tumors compared with adjacent normal tissues. Functional assays demonstrate that *DDX11* knockdown inhibits cell proliferation, colony formation, and tumor growth, while restoring gemcitabine sensitivity. Mechanistically, proteomic analysis and co-immunoprecipitation reveal that the interaction of DDX11 with PARP1 leads to increased poly(ADP-ribosyl)ation (PARylation), which promotes DNA repair and drug resistance. Notably, combining gemcitabine with the PARP inhibitor olaparib has synergistic anti-tumor effects on resistant cells. These findings indicate that DDX11 contributes to GBC progression and chemoresistance by regulating PARP1-mediated PARylation and that targeting this pathway with PARP inhibitors may overcome gemcitabine resistance. This study provides new insights into GBC drug resistance mechanisms and suggests that combining conventional chemotherapy with PARP inhibition is a potential therapeutic strategy for resistant patients. The DDX11-PARP1-PARylation axis represents a promising target for improving GBC treatment outcomes, particularly in gemcitabine-resistant patients.

## Introduction

Gallbladder carcinoma (GBC) is a highly malignant tumor of the biliary system with a poor prognosis [ 1, 2] . Its incidence among gastrointestinal tumors is relatively low (< 2 per 100,000), with a higher prevalence in females than in males [3]. Globally, there are approximately 116,000 new cases and 85,000 deaths annually, predominantly in the Far East and South American regions [1]. Current gemcitabine-platinum combination chemotherapies have shown limited efficacy (< 30% response rate) [ 4, 5] , and the 5-year survival rate remains at approximately 18% [6], highlighting significant clinical challenges.

Moreover, chemoresistance represents a major therapeutic obstacle [7], classified as either acquired (recurrence after initial response) or intrinsic (primary nonresponse) [8]. Underlying mechanisms include drug efflux upregulation [9], metabolic reprogramming, target mutations, apoptosis evasion [10], epigenetic alterations [11], and microenvironment remodeling [ 12, 13] . Biomarker-guided personalized therapies may improve outcomes [ 8, 14] .

While genomic studies have characterized GBC molecular features [ 15– 18] , resistance mechanisms remain understudied. Similarly, there is a lack of reliable biomarkers for the diagnosis, treatment and prognosis of this disease [19]. Our study established gemcitabine-resistant cell lines and identified the DNA helicase DDX11 as a potential resistance mediator through microarray analysis. As a genome stability regulator [ 20, 21] , DDX11 dysfunction may impair DNA repair, promoting chemoresistance, a novel finding that offers therapeutic insights.

The 2010’s discovery linking DDX11 mutations to Warsaw breakage syndrome revealed its crucial role in genomic stability and DNA damage repair [22]. DDX11 is a member of the DEAD/H-box family of ATP-dependent DNA helicases that was initially characterized through genetic screens for chromosome segregation mutants. This helicase plays critical roles in maintaining genomic integrity, as evidenced by the finding that biallelic mutations in DDX11 cause Warsaw breakage syndrome, a genetic disorder characterized by chromosomal instability and defective sister chromatid cohesion [20]. Mechanistically, DDX11 interacts directly with the replication fork protein Timeless to promote replisome progression and maintain epigenetic stability, particularly at G-quadruplex structures and during sister chromatid cohesion [23]. Furthermore, emerging evidence has demonstrated that DDX11 collaborates with the Fanconi anaemia pathway components 9-1-1 complex and SMC5/6 complex to counteract PARP inhibitor cytotoxicity [24], highlighting its multifaceted roles in the DNA damage response and repair pathways. While the oncogenic role of DDX11 in melanoma prognosis [25], cervical [26]and hepatocellular carcinogenesis [27] has emerged, its involvement in GBC resistance was previously unknown. We demonstrated for the first time that DDX11 promotes chemoresistance by modulating PARP1-mediated PARylation and DNA repair capacity.

PARP1, a key repair protein, catalyzes PAR polymer formation to recruit repair factors. PAR homeostasis is critical for DNA repair [28]; its dysregulation causes damage accumulation. In addition to facilitating single-strand break repair, PARP1 facilitates the recruitment of BRCA1 to double-strand breaks [29].

In the present study, our experiments confirmed that DDX11 overexpression in GBC promotes proliferation and chemoresistance. Mechanistically, DDX11 interacts with PARP1 to regulate PARylation levels, enhancing its ability to repair DNA. Some case reports have demonstrated the efficacy of PARP inhibitors in gallbladder cancer [ 30– 32] . Finally, we propose a combination therapy strategy of gemcitabine and olaparib for the treatment of gallbladder cancer to provide an experimental basis for clinical improvement in the treatment of gallbladder cancer.

## Materials and Methods

### Patients and specimens

This study enrolled 48 GBC patients who underwent radical cholecystectomy as primary treatment at the Department of Biliary-Pancreatic Surgery, Renji Hospital affiliated with Shanghai Jiao Tong University School of Medicine. Formalin-fixed, paraffin-embedded (FFPE) tissue samples were used for immunohistochemical (IHC) analysis. Additionally, 48 pairs of GBC tissues and matched noncancer tissues were collected during surgery and immediately preserved in liquid nitrogen for subsequent total RNA extraction. The study protocol was approved by the Ethics Committee of Renji Hospital, Shanghai Jiao Tong University School of Medicine (Shanghai, China).

### Reagents and cell culture

The human GBC cell lines NOZ and OCUG-1 were obtained from the Japanese Collection of Research Bioresources Cell Bank (Osaka, Japan), while GBC-SD was purchased from the Cell Bank of Shanghai Institutes for Biological Sciences, Chinese Academy of Sciences (Shanghai, China). The SGC-996 and EH-GB-1 cell lines were kindly provided by the Eastern Hepatobiliary Surgery Hospital Institute (Shanghai, China). All the cell lines were authenticated and were regularly tested for mycoplasma contamination during the experiments. The cells were cultured under standard conditions (37°C, 5% CO _2_) with the following media: NOZ in William’s medium (GNM41250; Genom, Shanghai, China), SGC-996 in RPMI-1640 (SH30809.01B; HyClone, Logan, USA), and the remaining three GBC cell lines in high-glucose DMEM (11965-092; Gibco, Carlsbad, USA). All media were supplemented with 10% fetal bovine serum (10270; Gibco) and 1% penicillin/streptomycin (15140-122; Gibco). Human gallbladder epithelial cells (HGEpCs), derived from cholecystitis patients, were maintained in complete EpiCM (4101; ScienCell, San Diego, USA).

### Induction of gemcitabine resistance in GBC cell lines

NOZ and GBC-SD cell lines with optimal viability and low passage numbers were cultured in DMEM supplemented with 10% fetal bovine serum and dual antibiotics. For NOZ-GR cell line induction, the cells were initially cultured continuously with 25 nM gemcitabine for 5 days (with the medium replaced by medium containing the drug on day 3), followed by adjustment to 2 nM until colony formation occurred (with the medium changed every 3 days). When the cells reached 90% confluence, they were passaged via gradual escalation of the gemcitabine concentration to 10 nM. Successful induction was confirmed when cells demonstrated stable proliferation in the presence of 10 nM gemcitabine, with growth rates comparable to those of parental cells with minimal apoptosis. For GBC-SD-GR induction, the cells were first treated with 50 nM gemcitabine for 5 days (the medium was replaced on day 3) and then reduced to 5 nM until colony formation. During subsequent passages (at 90% confluence), the concentration was progressively increased to 25 nM. The cells that maintained stable proliferation at 25 nM were considered successfully induced with GBC-SD-GR. The entire induction process lasted 4–6 months to ensure resistance stability. The established resistant cell lines were subsequently maintained in conventional DMEM supplemented with 10% fetal bovine serum, and cryopreservation was performed every 3 passages.

### Western blot analysis

For SDS-PAGE gel preparation, separation gels (7.5%–12.5%) were prepared according to target protein molecular weights by mixing equal volumes of lower gel buffers A and B with a 10% polymerization accelerator, then being cast onto gel plates and sealed with 70% ethanol for 30 min. The stacking gel was similarly prepared and polymerized for 20 min after a 15-well comb was inserted. Electrophoresis was performed at a constant voltage of 150 V for approximately 50 min until the bromophenol blue reached the gel bottom via freshly prepared 1× running buffer. For protein transfer, PVDF membranes (5.5 cm ×8.5 cm) were activated in methanol for 2–3 min and equilibrated in transfer buffer along with filter papers (6 cm ×9 cm). The transfer “sandwich” was assembled in the following order: sponge-filter paper-membrane-gel-filter paper-sponge from the anode to the cathode, ensuring bubble-free contact between layers. Transfer was conducted at a constant 100 V (400 mA current limit) for 120 min. The antibody incubation procedure included the following steps: 1-h blocking with 5% skim milk/PBST (with shaking at 15–20 rpm), overnight primary antibody incubation at 4°C followed by three 10-min PBST washes, and 1-h incubation with an HRP-conjugated secondary antibody at room temperature with identical washing steps. For detection, equal volumes of ECL solutions A and B were mixed and applied to membranes for 1–2 min, and chemiluminescent signals were captured via the ChemiDoc™ XRS+ system (Bio-Rad, Hercules, USA) with initial autoexposure followed by parameter optimization on the basis of signal intensity. All buffers were freshly prepared, air bubbles were rigorously avoided during transfer, and antibody dilutions were performed following the manufacturer’s instructions. The primary antibodies used included those against DDX11 (sc-271711; Santa Cruz, Santa Cruz, USA), Chk1 (ABclonal, Wuhan, China), p21 (Cell Signaling Technology, Danvers, USA), PARP1 (ABclonal) and β-actin (ABclonal). The secondary antibodies used were HRP-labelled goat anti-rabbit IgG (H + L) and HRP-labelled goat anti-mouse IgG (H + L) (Beyotime, Shanghai, China).

### Quantitative real-time PCR

The primers used for real-time qPCR were designed with *GAPDH* as the internal control. The gene symbols *DDX11* and *GAPDH* were searched with the species selection set to “Human”. The obtained sequences were synthesized by Shanghai Huajin Biotechnology (Shanghai, China), via the PAGE purification method, and the lyophilized products were reconstituted into 100 mM primer stock solutions according to the manufacturer’s instructions for long-term storage at -20°C. The detailed primer sequences are provided in Supplementary Table S1. For real-time qPCR, a 20 μL reaction system was prepared on ice protected from light following the manufacturer’s protocol compatible with the StepOnePlus system and contained 10 μL TB Green Premix Ex Taq (Tli RNaseH Plus) (2×) (TaKaRa, Dalian, China), 0.4 μL PCR forward primer (10 μM), 0.4 μL PCR reverse primer (10 μM), 0.4 μL ROX reference dye (50×), 2 μL cDNA library, and 6.8 μL ddH _2_O. After the plate was sealed, gently mixed and briefly centrifuged, the samples were loaded into the StepOnePlus™ Real-Time PCR System (Thermo Fisher Scientific, Waltham, USA). The two-step amplification program consisted of initial denaturation at 95°C for 30 s, followed by 40 cycles of 95°C for 5 s and 60°C for 30 s. For initial primer validation, a melt curve analysis was performed: 95°C for 15 s and 60°C for 1 min, followed by a gradual temperature increase of 0.3°C every 15 s up to 95°C. The relative *DDX11* expression level in tumor tissues compared with adjacent normal tissues was determined via the 2 ^–ΔΔCT^ method.

### Immunohistochemical staining and evaluation

The gallbladder carcinoma tissues and adjacent non-tumor tissues were fixed in 10% formalin for 24 h, washed three times with PBS, dehydrated through an ethanol gradient, cleared in xylene, and embedded in paraffin. Serial 4-μm sections were floated in a water bath, baked at 60°C for 2 h, and stored for subsequent use. The immunohistochemical staining procedure was performed as follows: after deparaffinization in xylene and rehydration through a graded alcohol series, endogenous peroxidase activity was blocked with 3% H _2_O _2_. Antigen retrieval was performed via citrate buffer, followed by blocking with 10% goat serum. The sections were incubated with a primary antibody against DDX11 (1:100 dilution) at 4°C overnight and then with an HRP-conjugated secondary antibody at room temperature for 45–60 min. Color development was achieved with DAB substrate (monitored microscopically), followed by hematoxylin counterstaining for 60 s. After differentiation in acid alcohol, the sections were dehydrated, cleared, and mounted with resin medium for microscopic examination. All steps were interspersed with three PBS washes. The expression of DDX11 in the samples was detected via an anti-DDX11 antibody (sc-271711, 1:50 dilution; Santa Cruz).

### siRNA transfection

The siRNA sequences targeting *DDX11* are listed in Supplementary Table S2 and were synthesized by Shanghai Jima Pharmaceutical Technology Co., Ltd. (Shanghai, China). Cells were transfected with siRNA using RFect siRNA Transfection Reagent (11013; Bio-Tran, Changzhou, China). One day prior to transfection, cells were passaged into 6-well plates at a seeding density of 25%–35%, and were expected to reach approximately 95% confluency on the fourth day. For the first knockdown, 50 nM siRNA was used, while 100 nM siRNA was applied for the second knockdown, with a volume ratio of siRNA to RFect reagent of 1:2. For each knockdown, the required volume of RFect was added to 250 μL of Opti-MEM medium and incubated at room temperature for 5 min. The required volume of siRNA was then added to another 250 μL of Opti-MEM medium and gently mixed. The diluted siRNA was added dropwise to the incubated RFect reagent, gently mixed by flicking, and incubated at room temperature for 15 min. To avoid the effects of antibiotics and serum components on transfection efficiency and cell viability, each well to be treated was rinsed with PBS buffer. The incubated mixture was added dropwise to the corresponding wells, and Opti-MEM medium was supplemented to a total volume of 1.5 mL. The plates were then returned to the CO _2_ incubator for incubation. After 6 h, the medium was replaced by serum-containing medium (with 10% serum) without antibiotics. Following transfection, total RNA and proteins were extracted from a portion of the cells to verify gene silencing efficiency.

### Lentiviral plasmid construction

shRNA sequences targeting human *DDX11* were selected from the Broad Institute database ( https://portals.broadinstitute.org/gpp/public/), prioritizing high-scoring or validated sequences. The oligos (PAGE-purified, synthesized by Shanghai Huajin Biotech as shown in Supplementary Table S3) were reconstituted in DEPC water (100 mM stock, stored at –20°C). For annealing, a 50 μL mixture (43 μL ddH _2_O, 5 μL 10× NEB buffer 2, 1 μL each forward/reverse oligo) was subjected to gradient annealing (95°C for 4 min→70°C for 10 min→0.3°C/1.5 min × 150 cycles to room temperature). Concurrently, pLKO.1 was digested with *Eco*RI/ *Age*I, and the 7 kb fragment was gel extracted. The ligation (20 ng digested vector + 2 μL annealed oligos in 20 μL with T4 ligase) proceeded at 16°C for 4–16 h. Transformed DH5α cells were plated on Amp ^+^ agar, and positive clones (3–5) were verified by sequencing after miniprep. The correct constructs were expanded in 200 mL of LB, and high-purity lentiviral plasmids were prepared via the NucleoBond® Xtra Midi kit (740410.50; Macherey-Nagel, Düren, Germany).

### Lentiviral packaging and infection

Lentiviral packaging was performed via the 293T three-plasmid system (psPAX2 + pMD2.G + pLKO.1-shRNA). Eighteen hours before transfection, 293T cells in the logarithmic growth phase were seeded in 100-mm dishes at 30%–35% confluency. For transfection, 20 μL of Lipo293™ reagent was mixed with 400 μL of OptiMEM (System 1) (Thermo Fisher Scientific) and incubated at room temperature for 5 min. Three micrograms of psPAX2, 1 μg of pMD2.G and 6 μg of pLKO.1-shRNA were sequentially added to another 400 μL of OptiMEM (System 2). System 2 was slowly added to System 1, gently mixed and incubated at room temperature for 15 min. Moreover, the medium was replaced by 5.5 mL of OptiMEM before the transfection mixture was added, the mixture was mixed gently, and the mixture was placed in a CO _2_ incubator. After 6 h, the medium was replaced by 10 mL of complete medium containing 10% FBS. The viral supernatant was collected after 72 h, filtered through 0.45-μm membranes and stored at –80°C. For infection, gallbladder cancer cells in the logarithmic growth phase were seeded in 35-mm dishes or 6-well plates at 40% confluency, followed by 18 h of culture. A total of 1.5 mL of viral supernatant was mixed with 1 mL of complete medium and 2 μL of polybrene to replace the original medium. After 24 h, fresh complete medium was added for continued culture.

### CCK-8 assay

The CCK-8 cell proliferation assay evaluates cellular proliferative capacity by measuring mitochondrial metabolic activity in viable cells. The experiment was performed in 96-well plates, with NOZ cells seeded at 800 cells/well and GBC-SD cells seeded at 1500 cells/well. Six time points (D0–D5) were established, with five replicate wells per group. After 6 h of cell attachment (D0), 10 μL of CCK-8 reagent was initially added, followed by a 2-h incubation before the OD _450_ values were measured. Subsequent measurements were performed daily at the same time point for 5 consecutive days. The relative proliferation rate was calculated using D0 values as a baseline, and time-proliferation curves were plotted to visualize dynamic changes in cell proliferation. This method offers high sensitivity and operational simplicity, enabling accurate assessment of differences in proliferation capacity under various treatment conditions. The CCK-8 assay kit was purchased from Yeasen Biotech (Shanghai, China).

### MTT assay

MTT Assay detects viable cells by measuring the reduction of MTT (40201ES72; Yeasen Biotech) to insoluble purple formazan crystals by mitochondrial succinate dehydrogenase in living cells. The formazan crystals are dissolved in DMSO, and the absorbance at 590 nm (OD _590_) is measured. The OD value is proportional to the number of viable cells within a certain range. The assay is widely used for evaluating anticancer drug cytotoxicity due to its high sensitivity and efficiency. Typically performed in 96-well plates, a 10× MTT stock solution (5 mg/mL) is prepared in PBS, filter-sterilized (0.45 μm), and diluted 1:10 in medium to make the working solution. After trypsin/EDTA digestion, single-cell suspensions of NOZ/NOZ-GR (1000 cells/100 μL) or GBC-SD/GBC-SD-GR (1800 cells/100 μL) were prepared. A 10-point drug dilution series (including a zero-concentration control) with 5 replicates per concentration (70 wells total) was used. Cells were seeded at 100 μL/well and cultured overnight. On day 1, medium was replaced by drug-containing fresh medium (100–150 μL/well); fresh medium was replenished on day 4. On day 5, 100 μL of MTT working solution was added per well. After 2 h of incubation, formazan crystals were dissolved in 100 μL DMSO with gentle shaking. OD _590_ was measured, and cell viability (%) was calculated as follows: cell viability (%) = (mean OD _590_ of treated group/mean OD _590_ of DMSO control) × 100%. IC _50_ values were determined using standard curve interpolation in GraphPad Prism 8.

### Colony formation assay

A colony formation assay was used to evaluate the proliferative potential of individual cells, which fundamentally differs from the CCK-8 method for detecting proliferation rates. The specific procedures were as follows: NOZ cells (500/well) or GBC-SD cells (800/well) were seeded in 6-well plates with duplicate wells for each group. Fresh culture medium was refreshed every 2–3 days to maintain optimal culture conditions. After 5–7 days of culture, microscopic examination was performed, and the experiment was terminated when most cell clusters exceeded 50 cells (typically requiring 6–8 days for NOZ cells and 10–14 days for GBC-SD cells). At termination, the cells were washed twice with PBS, fixed with 4% paraformaldehyde for 20 min (with shaking at 15–20 rpm), washed three times with PBS, and then stained with 0.5% crystal violet for 20 min (with shaking at 15–20 rpm). After staining, the wells were rinsed under running water, air-dried at room temperature, and finally scanned to record the results. The number of colonies was quantitatively analyzed via ImageJ software (National Institutes of Health, Bethesda, USA).

### Co-immunoprecipitation (Co-IP)

Total protein extraction was performed as follows: when NOZ and GBC-SD cells reached 90% confluency, they were washed twice with ice-cold PBS containing 1 mM PMSF, scraped, and centrifuged at 500 *g* (4°C, 3 min). For the experiments, 2 × 100 mm dishes of NOZ cells or 3 × 100 mm dishes of GBC-SD cells were used. Fresh NTEN lysis buffer (containing protease inhibitors, phosphatase inhibitors, and PMSF) was added (600 μL per tube), followed by rotation lysis at 4°C for 30 min. After centrifugation (14000 *g*, 4°C, 15 min), the supernatants were collected for BCA protein quantification. Protein samples (100 μg for Input, 500 μg for Co-IP) were prepared. For Co-IP, 50 μL of protein A/G magnetic beads were washed twice with 1 mL of NETN buffer. Antibody-bead complexes were prepared by incubating beads with 10 μL of DDX11 antibody (D2) or 2 μL of PARP1 antibody in 200 μL of NETN buffer (room temperature, 15–30 min, rotation), followed by 3 washes using NETN buffer. Protein samples (500 μg) were incubated with antibody-bead complexes overnight at 4°C. After 3 washes using NETN buffer, the complexes were denatured in 50 μL of 1× SDS-PAGE loading buffer (95°C, 10 min) and stored at –80°C.

### Tandem affinity purification technology combined with liquid chromatography-mass spectrometry analysis

NOZ cells were transfected with either pIRES2-SFB-vector or pIRES2-SFB-DDX11 plasmids and cultured for 72 h. After PBS washing, cells were scraped and centrifuged at 400 *g* for 5 min at 4°C. Cell lysis was performed using NETN buffer containing inhibitors with rotation for 30 min at 4°C. Lysates were centrifuged at 14,000 *g* for 15 min at 4°C, and supernatants were collected for protein quantification. For every 5 mg of protein, 30 μL of Flag M2 beads were washed with NETN buffer and incubated with samples overnight at 4°C. The beads were washed and eluted twice with Flag peptide (2 mg/mL), collecting 200 μL of eluate. Eluates were incubated with S protein agarose (69704; Novagen, Boston, USA) beads for 4–6 h at 4°C, followed by washing. Beads were boiled with 2× SDS loading buffer, and samples were separated by SDS-PAGE. Gels were silver-stained using a Pierce™ kit (24600; Thermo Fisher Scientific), imaged, and relevant bands were excised for LC-MS/MS analysis by Shanghai Zhongke New Life Biotech (Shanghai, China).

### Xenograft animals

Male nude mice (4 weeks old, weighing 18–20 g) were obtained from the Shanghai Laboratory Animal Center, Chinese Academy of Sciences (Shanghai, China). The animal experimental protocol was designed and implemented in strict accordance with the National Guidelines for Laboratory Animals. Nude mice were randomly divided into 2 groups (5–6 mice per group) and ear-tagged for identification. A 1-mL insulin syringe was used to subcutaneously inoculate NOZ cells stably transfected with shDDX11-1 into the right axilla of the mice, with a cell concentration of 600,000 viable cells per 100 μL of PBS. The mice were monitored for 20 min post-injection for adverse reactions. Tumor growth was tracked weekly by measuring the longest (a) and shortest (b) diameters (in mm) via Vernier callipers. The experiment was terminated if any tumor reached 20 mm in diameter, at which point all the mice were euthanized for tumor dissection, measurement, and imaging. The tumor volume (V, mm³) was calculated as follows: V = 1/2 × a × b ^2^.

### Combination therapy in nude mouse xenografts

A 100 μL phosphate-buffered saline suspension containing 600,000 NOZ-GR cells was subcutaneously injected into the axillary region of each nude mouse. The mice were monitored for 20 min post-injection for any adverse reactions. Gemcitabine was prepared in a vehicle solution containing 10% dimethylacetamide (DMA), 20% sulfobutyl-β-cyclodextrin, and 70% normal saline. Olaparib was dissolved in 5% DMA, 20% sulfobutyl-β-cyclodextrin, and 75% normal saline. The dosing regimens were as follows: gemcitabine: 25 mg/kg, twice weekly; intraperitoneal injection; olaparib: 40 mg/kg, once daily; and oral gavage. Treatment was initiated when the average tumor volume reached 120 mm³, with the mice randomly allocated into four groups. Tumor growth and body weight were monitored three times weekly and the tumor volume was calculated. The mice were humanely euthanized when the tumor volume reached 2000 mm³.

### Statistical analysis

The western blot bands were quantified via Image Studio Lite (v5.2). The signal intensities of DDX11 and PAR (analyzed at 100-250 kDa) were normalized to those of internal controls (GAPDH/β-actin) and calculated as fold changes versus controls. All experiments were independently repeated three times, and data are presented as the mean ± SD. Statistical analyses were performed via SPSS 24.0 and GraphPad Prism 8. For group comparisons, independent *t* tests (mRNA expression in gallbladder cancer vs adjacent tissues) or Friedman tests (western blot results) were used, with significance set at *P*  < 0.05. The mass spectrometry proteomics data were subjected to Gene Ontology (GO)/Kyoto Encyclopedia of Genes and Genomes (KEGG) pathway enrichment analysis via Metascape ( https://metascape.org), and the results were visualized as bubble plots, where size indicates protein count and color represents the significance level.

## Results

### High expression of DDX11 is associated with human gallbladder carcinoma progression

**Figure 1:**
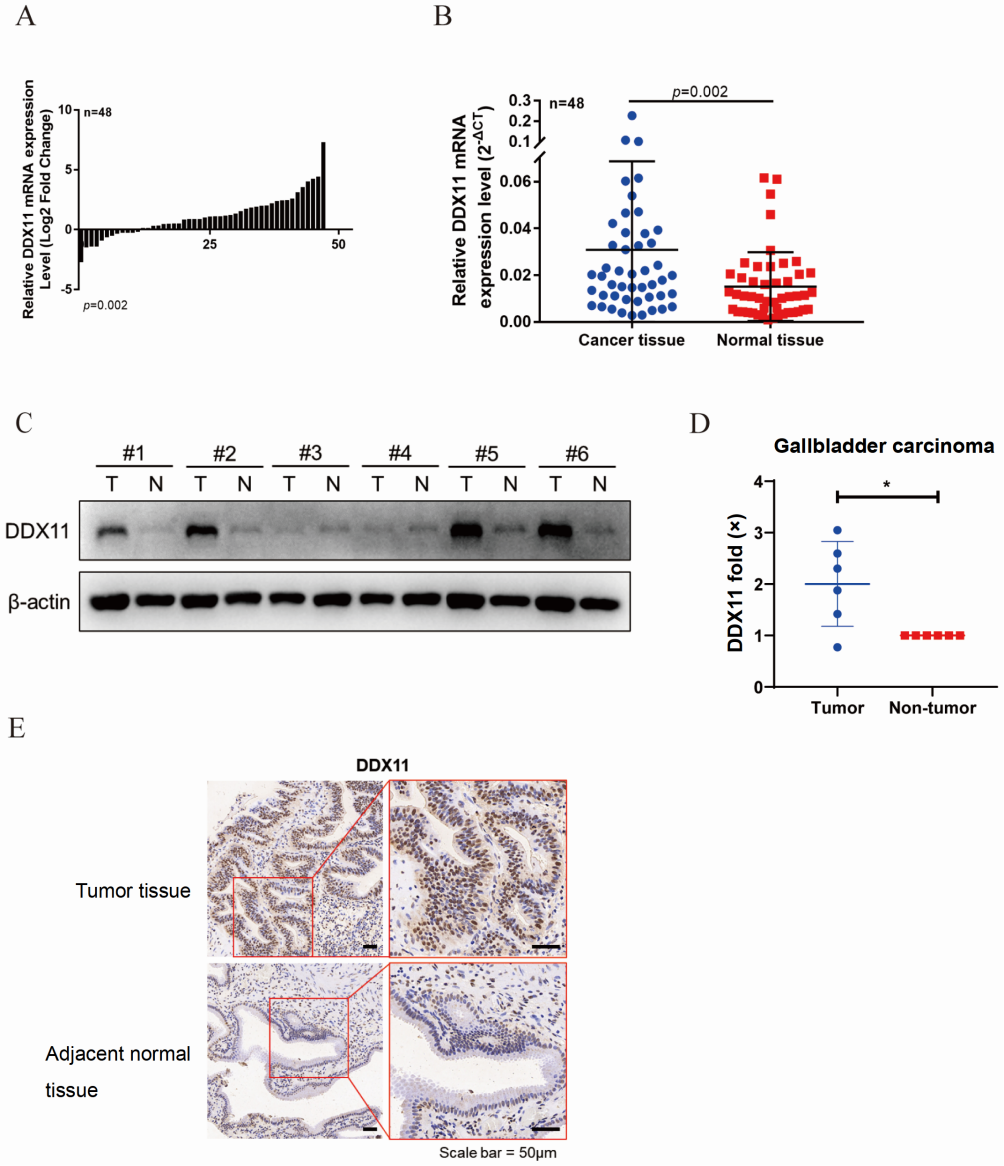
Figure 1 High expression of DDX11 is associated with human gallbladder carcinoma progression (A,B) High expression of DDX11 in gallbladder carcinoma. Total RNA was extracted from 48 pairs of gallbladder cancer tumor tissues and adjacent tissues, and the mRNA expression levels of DDX11 in gallbladder cancer tissues were detected by qRT-PCR. GAPDH is used as an internal reference. (C) Six pairs of gallbladder cancer tumor tissues and adjacent tissues were collected to extract total proteins, and the expression level of DDX11 in gallbladder cancer tissues was detected by western blot analysis. β-Actin was used as a loading control. (D) DDX11 band signal intensity was normalized to paired adjacent tissues to calculate the relative expression magnification of DDX11. The DDX11 bands were compared using the rank-sum test of the nonparametric Wilcoxon pairing data, *P < 0.05. (E) Immunohistochemical staining was used to detect the expression level of DDX11 in gallbladder cancer and adjacent normal tissue.

To investigate the clinical significance of DDX11 in human gallbladder cancer, we examined its mRNA expression in 48 paired tumor/adjacent tissues and detected significantly increased DDX11 expression in tumors ( Figure 1A,B). Western blot analysis of 6 paired tissues confirmed elevated protein levels ( Figure 1C,D). Immunohistochemistry further demonstrated the nuclear overexpression of DDX11 in tumor tissues ( Figure 1E). Similarly, the results revealed that DDX11 was more highly expressed in most tumor tissues than in their non-tumor counterparts. Therefore, DDX11 may play an important role in GBC progression.

### DDX11 promotes GBC cell proliferation and tumorigenesis *in vitro* and *in vivo*

**Figure 2:**
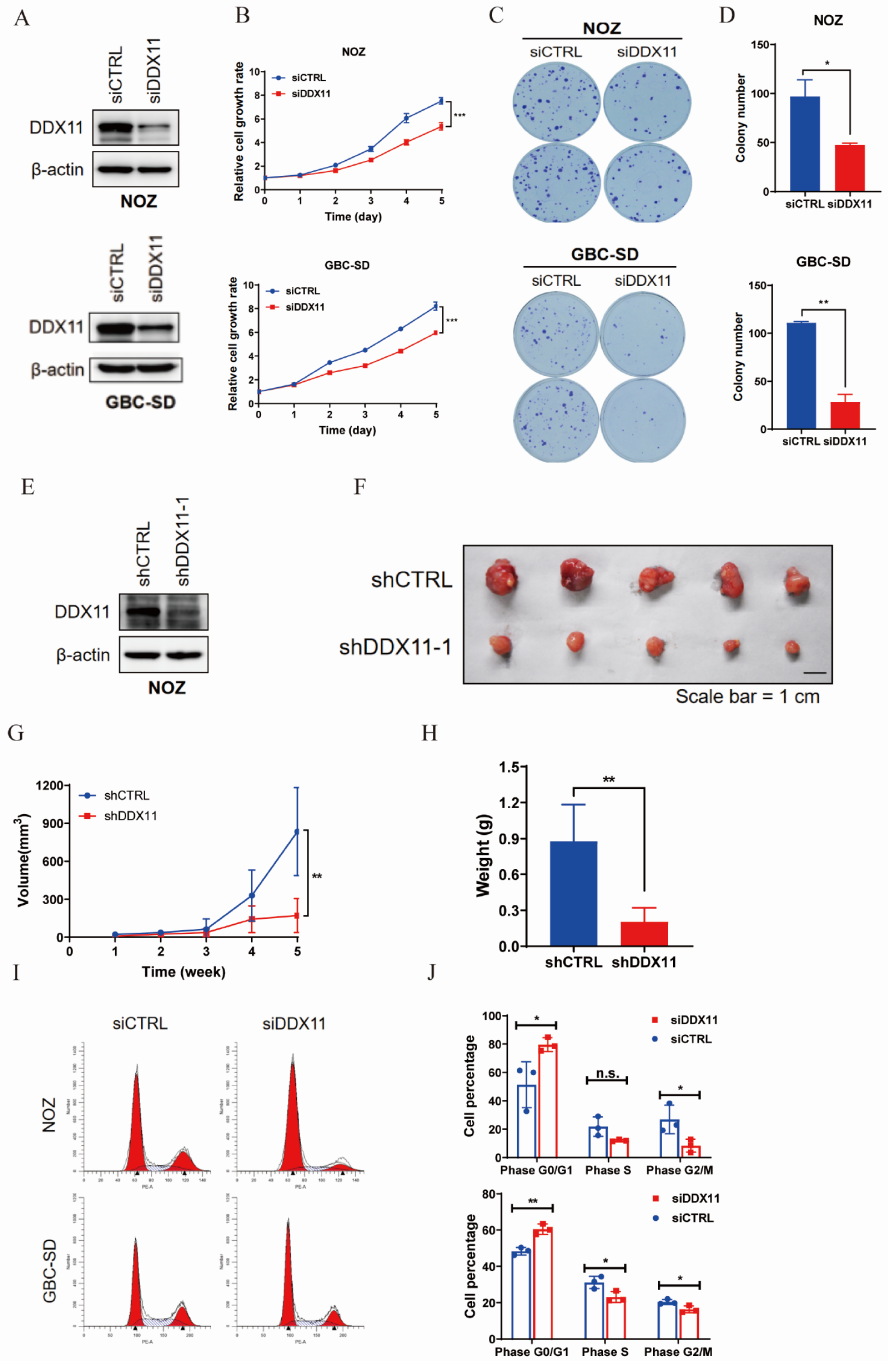
Figure 2 DDX11 promotes gallbladder cancer cell proliferation and tumorigenesis *in vitro* and *in vivo* (A) siRNA-mediated DDX11 silencing in NOZ and GBC-SD cells. (B) Cell proliferation assay was used to detect the effect of reduced DDX11 expression level on cell proliferation. (C) Colony formation assay was used to observe the effect of reduced DDX11 expression level on the cloning ability of cells. (D) Quantitative chart of the colony formation experiment. *P < 0.05, **P < 0.01, ***P < 0.001 (n = 3). (E) Analysis of the efficiency of DDX11 silencing induced by lentivirus in NOZ cells. (F–H) Stable NOZ-shDDX11-1 cells with low DDX11 expression and negative control cells were subcutaneously injected into naked mice, tumor length and short diameter were measured every 7 days to estimate the tumor volume and plot tumor growth curves (G), and mice were sacrificed approximately 40 days later, tumors were collected (F) and weighed (H). **P < 0.01. (I) Flow cytometric analysis of cell cycle distribution in NOZ and GBC-SD cells following DDX11 knockdown. (J) Statistical analysis of cell cycle distribution from three independent experiments (bar graph presentation). *P < 0.05, **P < 0.01; n.s., not statistically significant.

To investigate the role of DDX11 in gallbladder cancer cell proliferation and clonogenicity, we silenced *DDX11* in NOZ and GBC-SD cells via siRNA ( Figure 2A). CCK-8 assays demonstrated significantly impaired proliferation upon *DDX11* knockdown ( Figure 2B). Colony formation assays revealed that the silenced groups produced fewer than half the number of colonies produced by the controls ( Figure 2C,D). We introduced a highly effective shRNA (shDDX11-1) into NOZ cells using lentiviral delivery. After stable screening ( Figure 2E), NOZ-shDDX11-1 and control NOZ-shCTRL cells were subcutaneously injected into nude mice to establish xenograft models. Xenograft models revealed that the DDX11-low group had significantly smaller tumor volumes and masses than the control group did ( Figure 2F–H). Both *in vitro* and *in vivo* experiments consistently demonstrated the role of DDX11 in promoting gallbladder cancer cell proliferation and tumorigenicity. To investigate whether DDX11 regulates proliferation through cell cycle modulation, we silenced *DDX11* in NOZ and GBC-SD cells via siRNA and analyzed the cell cycle distribution via flow cytometry ( Figure 2I). The results demonstrated that *DDX11* knockdown significantly increased the proportion of G0/G1 phase cells (NOZ: 51.35% ± 13.24% vs 79.58% ± 3.95%; GBC-SD: 48.39% ± 1.69% vs 60.48% ± 2.38%) while markedly decreasing the proportion of G2/M phase cells (NOZ: 26.83% ± 8.27% vs 8.39% ± 3.74%; GBC-SD: 20.42% ± 1.13% vs 16.35% ± 1.57%) ( Figure 2J). These findings indicate that DDX11 promotes gallbladder cancer cell proliferation by alleviating G0/G1 arrest and facilitating cell cycle progression into the mitotic phase.

### DDX11 induces resistance to gemcitabine in GBC

To explore the mechanism of chemotherapy resistance to gemcitabine in GBC, we investigated the molecular mechanism of drug resistance by inducing the culture of drug-resistant cell lines and comparing their biological differences with those of parental cells.

**Figure 3:**
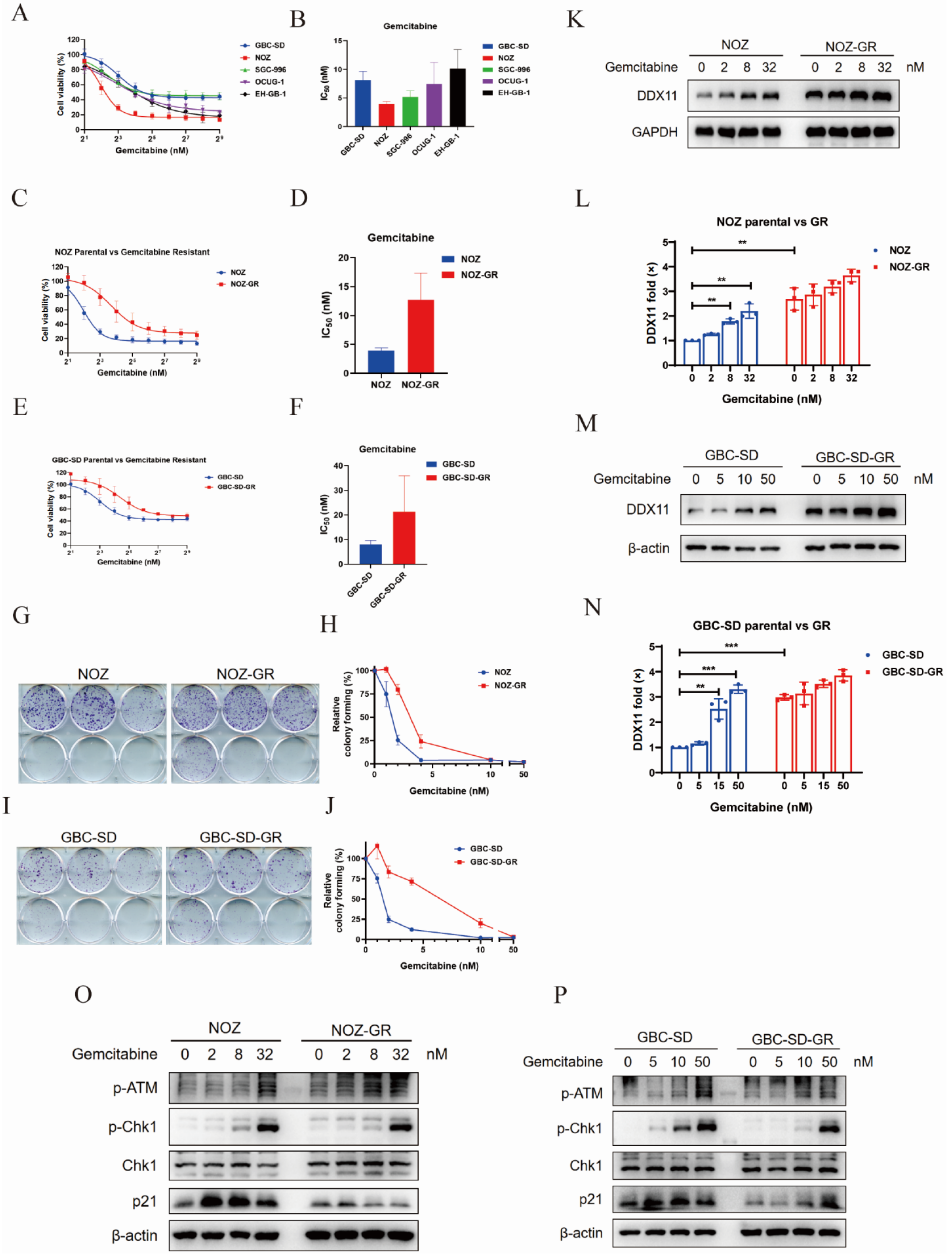
Figure 3 DDX11 induces resistance to gemcitabine in gallbladder cancer (A) Survival curve of gallbladder cancer cells based on MTT test. (B) Half-maximal inhibitory concentration (IC50) of gemcitabine in five gallbladder cancer cell lines. (C,E) Cell survival curves of gallbladder cancer resistant cell lines NOZ-GR (C), GBC-SD-GR (E) and corresponding parental cell lines from the MTT assay. (D,F) Gallbladder cancer resistant cell lines NOZ-GR (D), GBC-SD-GR (F) and gemcitabine IC50 of the corresponding parental cell line. (G,I) Colony formation of gallbladder cancer resistant cell lines NOZ-GR (G), GBC-SD-GR (I) and corresponding parental cell lines under different concentrations of gemcitabine. (H,J) The negative control group (DMSO treatment) was set to 100% clone formation rate, and the clone formation survival rate of the rest of the groups was calculated, and the relative clone formation capacity was plotted as a line chart. (K,L) Expressions of DDX11 in NOZ-GR and NOZ cells and after stimulation with different concentrations of gemcitabine. GAPDH was used as the internal reference, and the western blot bands were histogrammed based on the intra-group reference and the control group converted difference magnification. (M,N) Expression of DDX11 in GBC-SD-GR and GBC-SD cells and after stimulation with different concentrations of gemcitabine. GAPDH or β-actin was used as internal references, and the western blot bands were histogrammed based on the intra-group reference and the control group conversion difference magnification. **P < 0.01, ***P < 0.001. Statistics are derived from three independent experiments. (O) NOZ-GR and NOZ cells were stimulated with different concentrations (0, 2, 8, or 32 nM) of gemcitabine for 16 h, and total cellular protein was extracted for western blot analysis. β-Actin was used as a loading control. (P) GBC-SD-GR and GBC-SD cells were stimulated with different concentrations (0, 5, 15, or 50 nM) of gemcitabine for 16 h, and the total protein of the cells was extracted for western blot analysis. β-Actin was used as a loading control.

Five commonly used GBC cell lines (GBC-SD, SGC-996, NOZ, OCUG-1, and EH-GB-1) were selected and treated with different concentrations of gemcitabine (0–512 nM) for 5 days to detect cell viability. The results revealed that fast-proliferating cells were more sensitive to the drug and presented lower IC _50_ values ( Figure 3A,B). NOZ and GBC-SD cells with significant differences in IC _50_ values were selected to induce drug-resistant strains NOZ-GR and GBC-SD-GR. The IC _50_ of the resistant strain was significantly greater than that of the parental strains (NOZ-GR: 12.74 nM vs NOZ: 3.92 nM; GBC-SD-GR: 21.24 nM vs GBC-SD: 8.132 nM) ( Figure 3C–F). Colony formation experiments revealed that the colony formation of resistant strains was the strongest at drug concentrations ranging from 2–4 nM ( Figure 3G–J). CCK-8 detection revealed that there was no significant difference in the proliferation rate between drug-resistant strains and parental cells ( Supplementary Figure S1A,B).

Western blot analysis revealed that DDX11 was highly expressed in drug-resistant strains. After drug stimulation, the expression of DDX11 in parental cells (NOZ and GBC-SD) significantly increased. The expression of DDX11 in drug-resistant strains (NOZ-GR and GBC-SD-GR) was significantly greater than that in parental cells in the absence of drugs. These results showed that gemcitabine promoted the expression of DDX11 and that DDX11 was continuously expressed at high levels in the drug-resistant strains ( Figure 3K–N). As a pyrimidine anti-tumor drug, gemcitabine’s main metabolite can be incorporated into DNA double strands after entering cells, mainly by acting on the G1/S phase of the cell division cycle [33] and causing varying degrees of DNA damage, thereby activating the cellular DNA damage repair process. To further explore the differences in biological functions and related mechanisms between acquired gemcitabine-resistant cell lines and parental cells, we stimulated gallbladder cancer-resistant cell lines and parental cells with different concentrations of gemcitabine to observe their ability to repair DNA damage. Phosphorylated ATM protein (Ser1981) [34] and phospho-Chk1 (Ser345) [ 35, 36] are key pathway molecules through which cells initiate DNA damage repair after DNA double-strand breaks, and their expression levels correlate with the degree of DNA damage produced by cells. Activated CHK1 and p21 proteins act as cell cycle checkpoints, blocking the completion of mitotic progression. Therefore, we compared the degree of the DNA damage response produced by drug-resistant cell lines and parental cell lines under different drug concentration stimulation conditions ( Figure 3O,P). The results showed that in both drug-resistant and parental gallbladder cancer cells, the cells produced a DNA damage stress response and initiated the DNA repair process after gemcitabine treatment. However, the expression levels of markers of DNA damage stress in cells were relatively low in response to the same stimulation conditions in drug-resistant strains, and cells presented strong DNA damage repair ability and were not susceptible to cell cycle checkpoint-induced mitotic process arrest. Therefore, drug-resistant gallbladder cancer cell lines are strongly resistant to DNA damage stress and have strong DNA damage repair ability.

### DDX11 interacts with PARP1 and is involved in the process of PARylation

**Figure 4:**
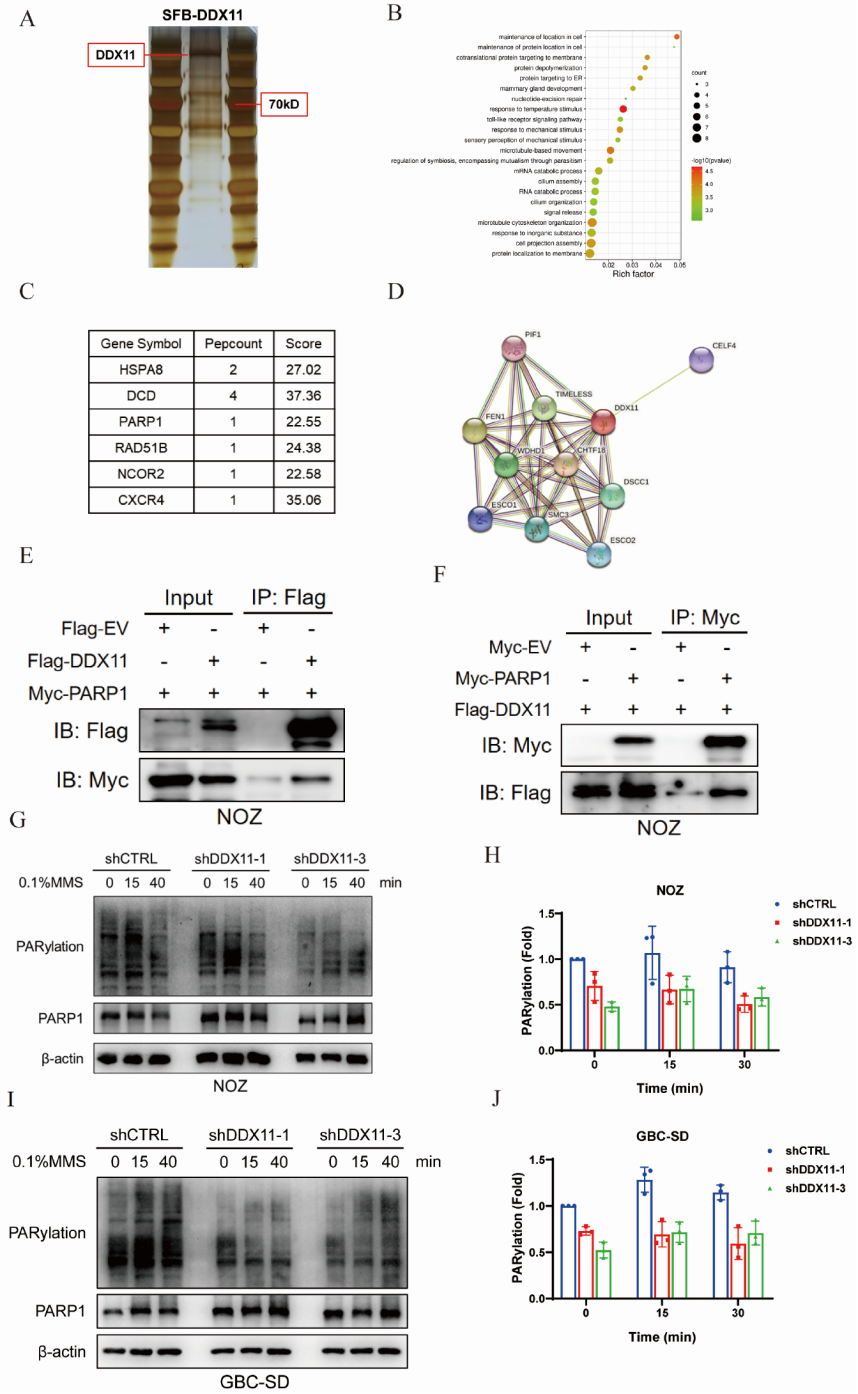
Figure 4 DDX11 interacts with PARP1 and is involved in the process of PARylation (A) Tandem affinity purification of protein molecules interacting with DDX11, samples electrophoretic migration in 4%-20% gradient gel, a rapid silver staining experiment was then performed to observe the protein bands. (B) Metascape online database enrichment analysis of cell biology pathways involved in the interacting protein network, and a bubble map was plotted, with the gene enrichment index on the abscissa and the pathway name or description on the ordinate. The size of the bubble represents the number of interacting proteins involved in the pathway, and the color of the bubble represents the significance of the test. (C) Excerpts from liquid chromatography-mass spectrometry proteomics analysis. (D) STRING online database retrieved with DDX11 interacting protein network. (E) Seventy-two hours after the exogenous expression of Flag-DDX11 and Myc-PARP1 in NOZ cells, the total protein of the cells was extracted and co-incubated with Flag M2 affinity gel for immunoprecipitation experiments, and the co-precipitated protein samples were subjected to western blot analysis with the corresponding tag antibodies. (F) Seventy-two hours after the exogenous expression of Myc-PARP1 and Flag-DDX11 in NOZ cells, the total protein was extracted and co-incubated with anti-Myc magnetic beads for immunoprecipitation experiments, and the co-precipitated protein samples were subjected to western blot analysis with the corresponding tag antibodies. (G,I) Lentivirus-induced NOZ (G) and GBC-SD (I) cells with stable DDX11 silencing were treated with 0.1% MMS for 40 min, and the total protein was extracted and detected by western blot analysis to detect intracellular PARP1 and PAR expression levels. β-Actin was used as a loading control. (H,J) The signal intensity of PAR in NOZ (H), GBC-SD (J) cells in the treatment group was normalized to the β-actin signal intensity in the group, and then compared to the negative control group (1.0-fold). Bar chart statistics are derived from three independent experiments, and relative magnifications are expressed as the mean ± SD.

As the main executors of life activities, proteins often function through protein interactions or the formation of protein complexes. DDX11 is a DNA helicase with ATPase activity that functions by interacting with a variety of proteins during cell division and DNA replication [ 37– 39] , but there is a lack of direct evidence indicating that DDX11 is involved in DNA damage repair through the formation of protein complexes. Therefore, the aim of this study was to systematically investigate whether the DDX11-interacting protein network contains DNA damage repair-related proteins. To obtain highly specific experimental results, we used tandem affinity purification technology combined with liquid chromatography-mass spectrometry analysis to construct a DDX11-interacting protein network. Specifically, we first constructed the pIRES2-SFB-DDX11 recombinant plasmid to obtain the interacting protein complex via two-step affinity purification of the Flag tag and S protein. SDS-PAGE (4%‒20% gradient gel) and rapid silver staining revealed obvious protein bands at multiple molecular weight intervals, with the exception of the DDX11 band ( Figure 4A). Mass spectrometry identified more than 120 potential DDX11-interacting proteins. We uploaded the list of protein-coding genes to the Metascape online database for pathway enrichment analysis and found that these interacting proteins were significantly enriched in several important biological pathways ( Figure 4B), and some of the interacting protein molecules were selected on the basis of the enriched cell biology pathways ( Figure 4C). In addition, we searched the human DDX11-interacting protein network ( Figure 4D) in the Search Tool for the Retrieval of Interacting Genes/Proteins (STRING) online database ( https://string-db.org). Most of the interacting protein networks listed in the database are involved in cell division and genetic material replication, and there was no prediction of DNA damage repair. However, our pathway analysis unexpectedly revealed that DDX11-interacting proteins, such as PARP1 and RAD51B, contain known key molecules for DNA damage repair. Combined with the results of previous experiments, DDX11 may be involved in DNA damage repair and enhance the tolerance of tumor cells to DNA damage; thus, we focused on the association between DDX11 and the DNA damage repair pathway. Coimmunoprecipitation experiments confirmed the direct interaction of DDX11 with PARP1 in NOZ gallbladder cancer cells ( Figure 4E,F). As a core regulator of DNA damage repair, PARP1 is involved in a variety of repair pathways, including base excision repair, single-strand break repair, and double-strand break repair (homologous recombination and nonhomologous end joining) [40]. Notably, PARP1 serves as both the principal executor and major substrate of poly-ADP ribosylation (PARylation), a crucial posttranslational modification that mediates the DNA damage response through the transfer of ADP-ribose units from NAD ^+^ to acceptor proteins. To explore whether DDX11 regulates the PARP1-mediated PARylation process, we examined the dynamic changes in PARylation in *DDX11*-knockdown cells after treatment with the alkylating agent MMS. The results revealed that the level of PAR in the control group (shCTRL) increased rapidly after 0.1% MMS treatment, peaked at 15 min and then gradually decreased. In contrast, stable *DDX11* knockdown (shDDX11-1/3) not only significantly reduced the basal PAR level but also significantly reduced the amplification of PARylation after MMS induction ( Figure 4G–J). Importantly, *DDX11* knockdown did not affect the expression level of the PARP1 protein, indicating that DDX11 affects the PARylation process by regulating the enzymatic activity of PARP1 rather than its expression level. Taken together, these results suggest that DDX11 is involved in regulating DNA damage-induced PARylation by interacting with PARP1 and that downregulation of DDX11 expression leads to impaired PARylation in cells, which may affect the efficiency of DNA damage repair.

### DDX11-PARP1 interaction and PARylation mediate gemcitabine resistance in GBC

**Figure 5:**
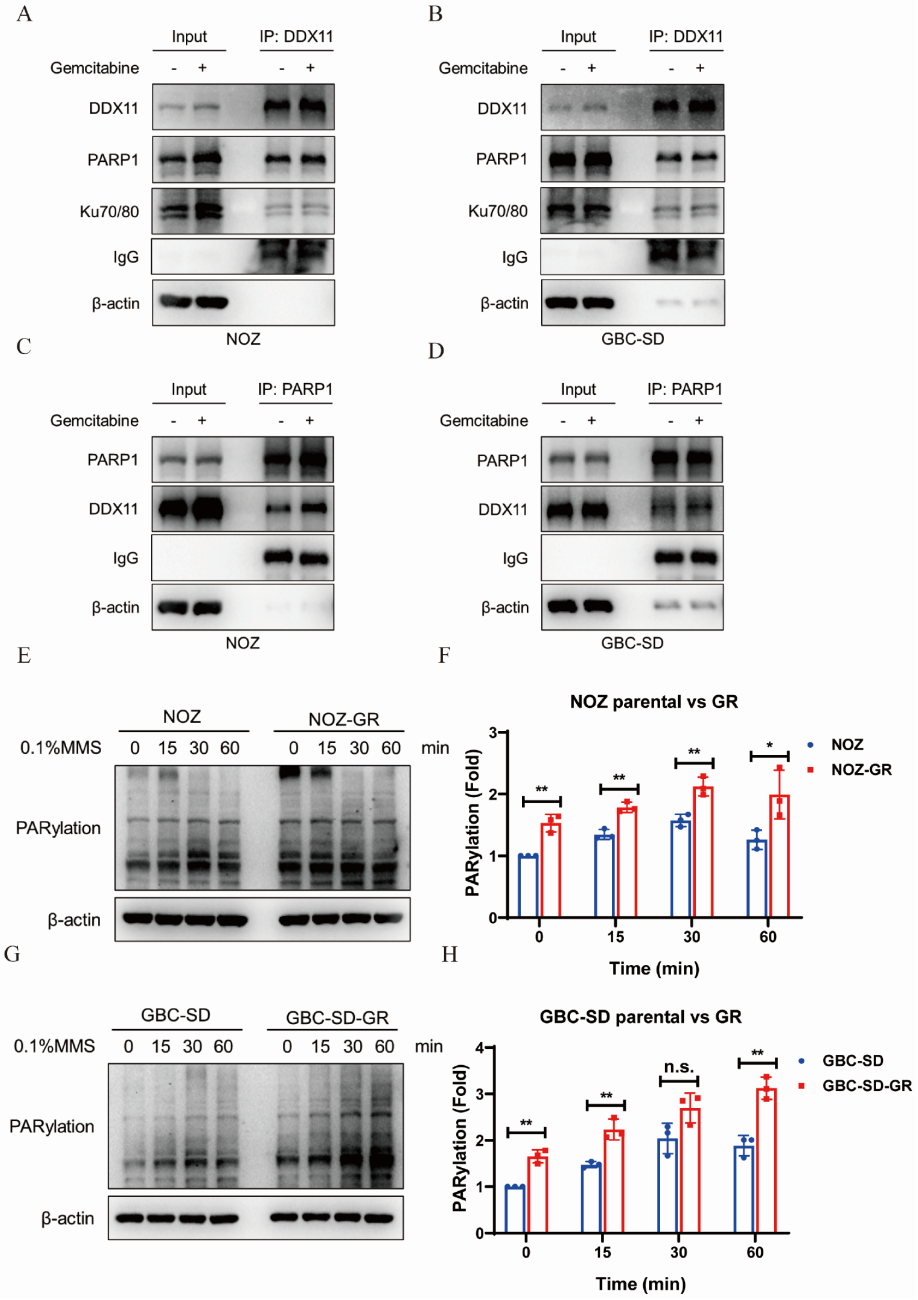
Figure 5 DDX11-PARP1 interaction and PARylation mediate gemcitabine resistance in gallbladder cancer (A,B) NOZ (A) and GBC-SD (B) cells were treated with gemcitabine (NOZ: 250 nM; GBC-SD: 400 nM) for 14 h, total cellular protein was extracted and co-incubated with DDX11 antibody for immunoprecipitation. The co-precipitated samples were subjected to western blot analysis by corresponding antibodies. β-Actin and IgG were used as internal references and controls. (C,D) NOZ (C) and GBC-SD (D) cells were treated with gemcitabine (NOZ: 250nM; GBC-SD: 400 nM) for 14 h, total cell protein was extracted, co-incubated with PARP1 antibody for co-immunoprecipitation, and the co-precipitated sample was subjected to western blot analysis with the corresponding antibody. β-Actin and IgG were used as internal references and controls. (E,G) Gemcitabine-resistant cell lines NOZ-GR (E) and GBC-SD-GR (G) and parental cells were treated with 0.1% MMS for 60 min, and the total protein was extracted and the level of intracellular PARylation was detected by western blot analysis. β-Actin was used as a loading control. (F,H) The signal intensities of PAR in the resistant cell lines NOZ-GR (F) and GBC-SD-GR (H) were normalized relative to the β-actin signal intensities and then converted to the parental cell group (1.0-fold). Bar chart statistics are derived from three independent experiments, and relative magnifications are expressed as mean ± SD. *P < 0.05, **P < 0.01; n.s., not statistically significant.

Previous experiments have shown that DDX11 expression is affected by gemcitabine treatment ( Figure 3K–N), which prompted us to further investigate whether this drug affects the interaction of DDX11 with PARP1. In NOZ and GBC-SD cells, 14 h of gemcitabine treatment slightly increased the interaction of DDX11 with PARP1 ( Figure 5A–D). Moreover, we examined other key molecules involved in DNA damage repair, such as Ku70/80, and found that DDX11 also interacted with this molecule ( Figure 5A,B), which provides direct evidence for the involvement of DDX11 in DNA damage repair. Further studies revealed that gemcitabine-resistant cell lines (NOZ-GR and GBC-SD-GR) have unique DNA damage response characteristics. Compared with parental cells, drug-resistant cells not only have higher basal DDX11 expression levels but also exhibit a lower DNA damage response (DDR) and repair-related molecular expression levels after the same gemcitabine treatment. To further understand this phenomenon, we compared the dynamics of PARylation in drug-resistant cells with those in parental cells in response to MMS-induced acute DNA damage. The results revealed that the basal PARylation level of drug-resistant cells was significantly greater than that of parental cells and that the PARylation level of parental cells typically peaked after 30 min and then decreased after MMS treatment, whereas drug-resistant cells maintained a stable high level of PARylation ( Figure 5E–H). These findings suggest that the DNA damage repair capacity of drug-resistant cells may be increased by maintaining increased PARylation activity, thereby increasing resistance to chemotherapeutic agents.

### The combination of gemcitabine and olaparib has synergistic anti-tumor effects on gemcitabine-resistant GBC cell lines

PARP inhibitors, particularly third-generation agents that target PARP1 and other PARP family members, have achieved widespread clinical application. These inhibitors have advanced to phase III clinical trials for treating BRCA2-mutated breast and ovarian cancers, leveraging the synthetic lethality between PARP1 deficiency and BRCA2 deficiency [40]. Substantial evidence supports the therapeutic potential of PARP inhibitors across various tumors with DNA repair defects, primarily through the suppression of PARP1 catalytic activity and subsequent PARylation [41].

**Figure 6:**
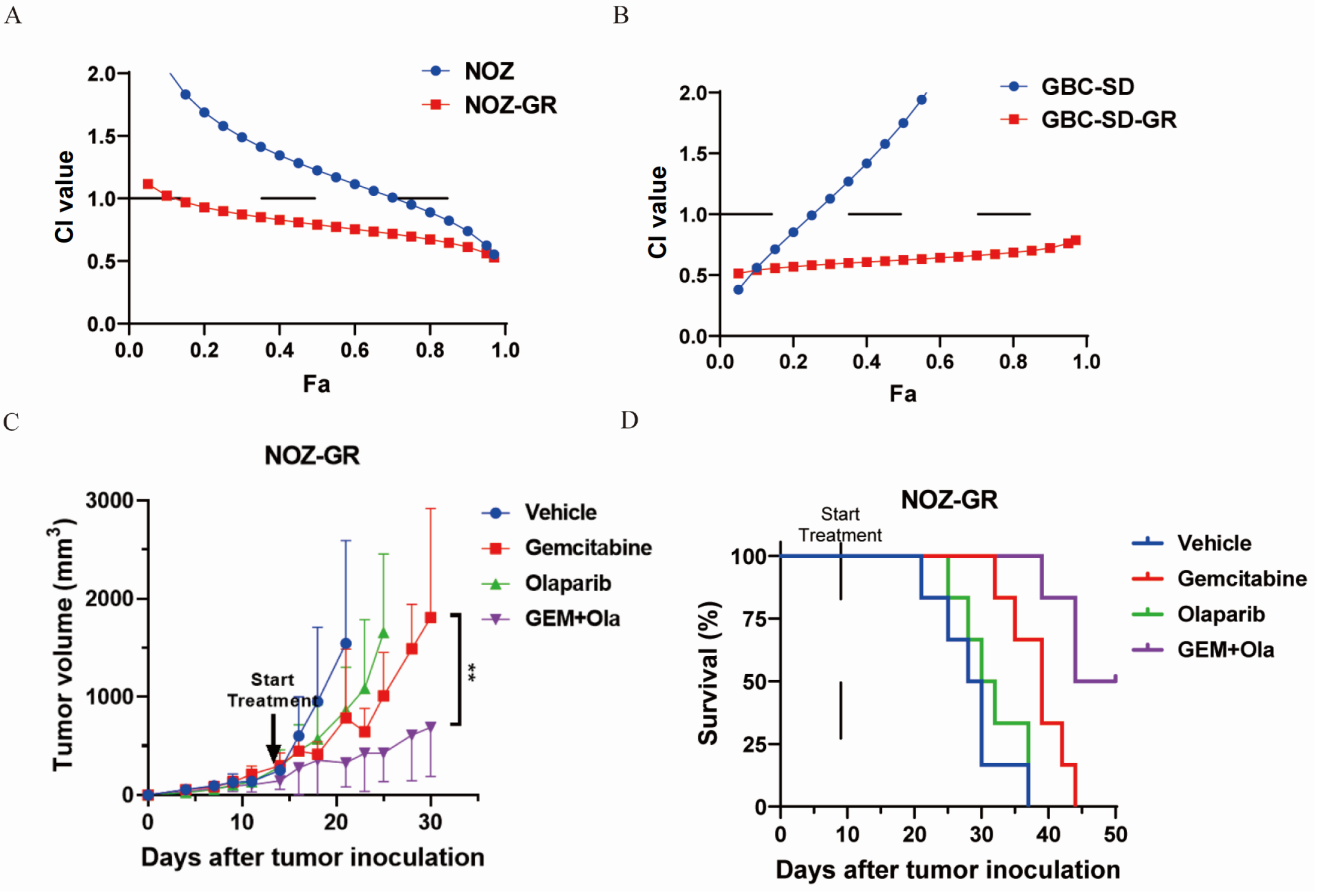
Figure 6 Gemcitabine synergizes with olaparib in suppressing gallbladder cancer cells *in vitro* and *in vivo* (A,B) The viability of gemcitabine-resistant gallbladder cancer cell lines NOZ-GR and their parental cells (A), as well as GBC-SD-GR and their parental cells (B), was assessed by MTT assay after 96 h of treatment with gemcitabine and/or olaparib. The combination index (CI) of the two drugs in gallbladder cancer cells was calculated. (C) Subcutaneous tumors of NOZ-GR in nude mice (n = 6) were treated with gemcitabine and/or olaparib. Tumor volume was measured periodically and analyzed by ANOVA. Data are presented as the mean ± SD; **P < 0.01. (D) Growth curve analysis of the NOZ-GR animal model.

Our findings demonstrate that gemcitabine-resistant cell lines exhibit increased DDX11 expression and enhanced PARylation activity upon DNA damage. This prompted us to investigate the combinatorial effect of gemcitabine with the PARP inhibitor olaparib. Using MTT assays after 96 h of drug exposure, we quantified cell viability and calculated combination indices (CIs) ( Figure 6A,B). The combination had synergistic effects (CI < 1) on NOZ, NOZ-GR, and GBC-SD-GR cells but had antagonistic effects (CI > 1) at relatively high cytotoxicity levels (> 50%) in GBC-SD cells. Notably, both resistant lines presented lower CIs than their parental counterparts did, indicating superior synergistic tumoricidal activity in resistant phenotypes.

To validate these findings *in vivo*, we established NOZ-GR xenograft models in nude mice ( Figure 6C,D). Monotherapy with either agent resulted in limited tumor growth inhibition, whereas the combination regimen significantly suppressed tumor progression (gemcitabine vs combination, *P*  < 0.01) and prolonged survival ( Figure 6D). These results collectively demonstrate the robust synergistic anti-tumor efficacy of the combination of gemcitabine and olaparib against gemcitabine-resistant gallbladder cancer in both cellular and animal models.

## Discussion

GBC, the most prevalent malignancy of the biliary system, is facing increasing challenges related to drug resistance in clinical management. As a first-line chemotherapeutic agent, gemcitabine resistance has emerged as a critical limiting factor for GBC treatment efficacy [42]. Our team has thus far invested great efforts in research on gallbladder carcinoma [ 14, 43, 44] . We have achieved significant progress in elucidating the mechanisms underlying tumor proliferation [ 45, 46] , metastasis [ 47, 48] , and immune regulation [49] in gallbladder carcinoma.

In this study, we established gemcitabine-resistant cell models and, for the first time, revealed the pivotal role of DDX11 in GBC chemoresistance: DDX11 expression was markedly upregulated in resistant cells and could be further induced by drug stimulation. Functional investigations revealed that resistant DDX11-overexpressing cells presented increased DNA damage repair capacity and increased resistance to genotoxic stress. Clinical specimen analysis revealed significantly higher DDX11 expression levels in GBC tissues than in adjacent nontumor tissues.

Mechanistically, we not only confirmed the role of DDX11 in promoting GBC cell proliferation, clonogenicity and tumorigenesis but also, more importantly, identified its participation in regulating PARylation through direct interaction with PARP1. Elevated DDX11 expression in resistant cells substantially augmented the cellular DNA damage response by potentiating PARP1-mediated PARylation. Finally, we proposed a therapeutic strategy combining gemcitabine and olaparib for gallbladder cancer and validated their synergistic effects at both the cellular and animal levels. Previous studies have confirmed that the gemcitabine-olaparib combination regimen significantly enhances anti-tumor activity over single-agent therapy in pancreatic cancer [50].

Notably, further validation of the DDX11-PARP1 interaction via confocal microscopy and other approaches is warranted, along with a more comprehensive comparative analysis of DDX11 expression patterns in clinically resistant samples, which will constitute key directions for future investigations.

Currently, pharmacological inhibitors targeting PARP1 (and other PARPs) have been widely applied in clinical practice. Third-generation PARP inhibitors have fully entered phase III clinical trials for treating BRCA2-mutated breast and ovarian cancers, leveraging the synthetic lethality between PARP1 deficiency and BRCA2 deficiency to achieve tumor control [41]. Substantial evidence has demonstrated that PARP inhibitors can be used to treat various tumors with DNA repair gene defects. One of the pharmacological mechanisms of action of PARP inhibitors involves the suppression of PARP1 catalytic activity and the inhibition of PARylation levels [42]. While PARP inhibitors show promise in advanced cancer treatment, robust biomarkers are lacking. DDX11 has emerged as a potential candidate.

Overall, our study provides the first systematic elucidation of the molecular mechanism by which DDX11 regulates GBC chemoresistance through the PARP1-PARylation pathway, identifying novel therapeutic targets and combination strategies to overcome clinical chemotherapy resistance.

Nevertheless, the current study has certain limitations that should be noted. First, the interaction between DDX11 and PARP1 needs to be further confirmed by additional experimental evidence, such as confocal microscopy. Second, we lacked a comparative analysis of DDX11 expression levels in clinical samples from gemcitabine-sensitive versus gemcitabine-resistant gallbladder cancer patients. These aspects will be systematically investigated and improved in our follow-up studies to provide more comprehensive evidence supporting our conclusions.

## Supporting information

25650Supplementary_data

## Supplementary Data

Supplementary data are available at *Acta Biochimica et Biophysica Sinica* online.
