# Supplementary material for: DDX11 interacts with PARP1 to facilitate PARylation, thereby promoting gallbladder cancer progression and conferring gemcitabine resistance: DDX11 interacts with PARP1 to facilitate PARylation
Source: Acta Biochim Biophys Sin (Shanghai). 2025 Aug 26;58(5):1008–22. doi: 10.3724/abbs.2025155 (PMC13191460; doi:10.3724/abbs.2025155)
Supplement: 25650Supplementary_data [file 25650Supplementary_data.docx]

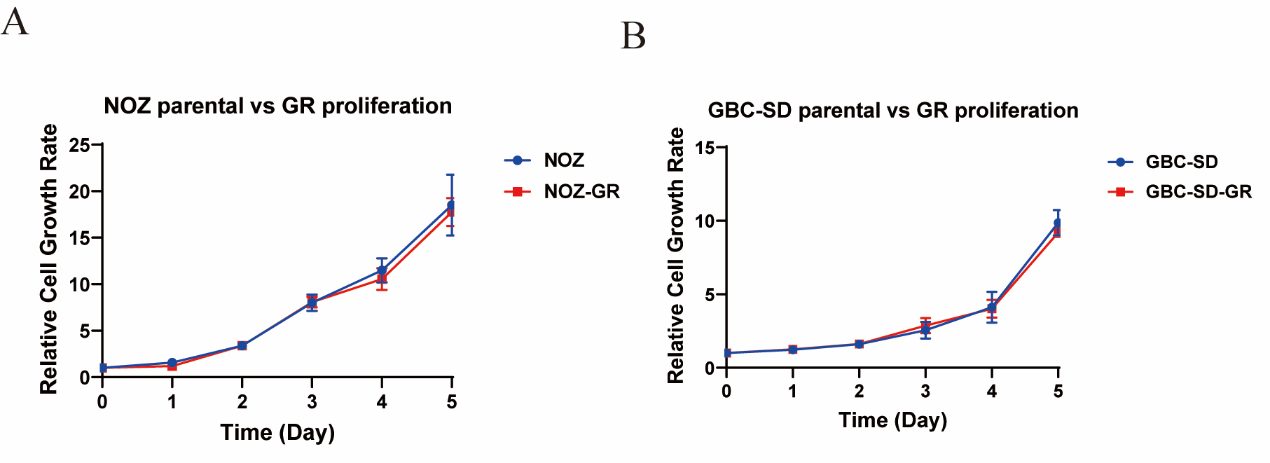


**Supplementary Figure S1. Relative proliferation rate of gemcitabine-resistant cells and parental cells** (A,B) Based on the CCK-8 assay to detect the proliferation rate of gemcitabine-resistant gallbladder carcinoma cell lines and parental cell lines, plot the amplification curve.

**Supplementary Table S1. The sequences of primers used in this study**

| *DDX11* | Forward primer | 5′-TCCTCGCCGAATACGAGAGT-3′ |
| --- | --- | --- |
|  | Reverse primer | 5′-GGAGTGTGTCCGACTACAGTAA-3′ |
| *GAPDH* | Forward primer | 5′-GGAGCGAGATCCCTCCAAAAT-3′ |
|  | Reverse primer | 5′-GGCTGTTGTCATACTTCTCATGG-3′ |

**Supplementary Table S2. siRNA sequences used in this study**

| siControl | 5′-TTCTCCGAACGTGTCACGT-3′ |
| --- | --- |
| siDDX11-1 | 5′-CCAACTGGCACTGGGAAGTCCTTAA-3′ |
| siDDX11-2 | 5′-CCCTTACATGATGAGAAAGAT-3′ |
| siDDX11-3 | 5′-CCTGTGTCTGTCTTCTTCCTGCGAA-3′ |

**Supplementary Table S3. shRNA sequences used in this study**

| Negative control-F | 5′-CCGGCAACAAGATGAAGAGCACCAACTC  GAGTTGGTGCTCTTCATCTTGTTGTTTTTG-3′ |
| --- | --- |
| Negative control-R | 5′-AATTCAAAAACAACAAGATGAAGAGCACCAA  CTCGAGTTGGTGCTCTTCATCTTGTTG-3′ |
| DDX11-shRNA-1-F | 5′-CCGGCCCTTACATGATGAGAAAGATCTC  GAGATCTTTCTCATCATGTAAGGGTTTTTG-3′ |
| DDX11-shRNA-1-R | 5′-AATTCAAAAACCCTTACATGATGAGAAAGAT  CTCGAGATCTTTCTCATCATGTAAGGG-3′ |
| DDX11-shRNA-2-F | 5′-CCGGGAGAGCTGCCTCAGATGATATCTC  GAGATATCATCTGAGGCAGCTCTCTTTTTG-3′ |
| DDX11-shRNA-2-R | 5′-AATTCAAAAAGAGAGCTGCCTCAGATGATAT  CTCGAGATATCATCTGAGGCAGCTCTC-3′ |
| DDX11-shRNA-3-F | 5′-CCGGCACTCTCTGGTCTCAATTTAACTC  GAGTTAAATTGAGACCAGAGAGTGTTTTTG-3′ |
| DDX11-shRNA-3-R | 5′-AATTCAAAAACACTCTCTGGTCTCAATTTAA  CTCGAGTTAAATTGAGACCAGAGAGTG-3′ |
